# A Cross-Sectional Pilot Study on Association of Ready-to-Eat and Processed Food Intakes with Metabolic Factors, Serum Trans Fat and Phospholipid Fatty Acid Compositions in Healthy Japanese Adults

**DOI:** 10.3390/nu16071032

**Published:** 2024-04-02

**Authors:** Chizuko Maruyama, Miya Uchiyama, Ariko Umezawa, Aoi Tokunaga, Akari Yasuda, Kanako Chibai, Chieko Fukuda, Rina Ichiki, Noriko Kameyama, Masakazu Shinohara

**Affiliations:** 1 Division of Food and Nutrition, Graduate School of Human Sciences and Design, Japan Women’s University, Tokyo 112-8681, Japan; m1622302om@gr.jwu.ac.jp; 2 Department of Food and Nutrition, Faculty of Human Sciences and Design, Japan Women’s University, Tokyo 112-8681, Japan; umezawaa@fc.jwu.ac.jp (A.U.); kameyaman@fc.jwu.ac.jp (N.K.); 3 The Integrated Center for Mass Spectrometry, Kobe University Graduate School of Medicine, Kobe 650-0017, Japan; mashino@med.kobe-u.ac.jp; 4 Division of Molecular Epidemiology, Kobe University Graduate School of Medicine, Kobe 650-0017, Japan

**Keywords:** processed food, ready-to-eat meal, odd-number fatty acid, trans fatty acid, elaidic acid, Japanese, liver function, fiber, EPA, DHA

## Abstract

Frequently consuming processed and ready-to-eat (RTE) foods is regarded as unhealthy, but evidence on the relationships with circulating metabolic parameters is lacking. Japanese residents of a metropolitan area, 20 to 50 years of age, were studied in terms of anthropometric and biochemical parameters, including circulating trans fat and serum phospholipid fatty acid levels. Processed foods, except drinks and dairy items, were categorized according to requirements for additional ingredients and cooking before eating. Processed and RTE foods were divided according to fat and/or oil content into non-fatty or fatty foods. The participants were grouped into tertiles based on the energy percent (En%) derived from fatty-RTE foods. Fatty-RTE En% showed negative associations with fish, soybean and soybean products, dairy, eggs, vegetables, seaweed/mushrooms/konjac, fruit and non-oily seasonings reflecting lower dietary fiber, eicosapentaenoic acid (EPA) and docosahexaenoic acid (DHA), and mineral and vitamin intakes, while the associations with fat/oil, confectionaries, and sweet beverages were positive. Fatty-RTE En% consumption was positively associated with alkaline phosphatase, leucine aminopeptidase, direct bilirubin, elaidic acid, and C18:2 but inversely associated with HDL cholesterol, C15:0, C17:0, EPA, and DHA. A higher fatty-RTE food intake was suggested to contribute to unbalanced nutrient intakes, as reflected in lipid metabolic parameters. Further large-scale studies are needed to evaluate the quality and impacts of RTE foods.

## 1. Introduction

Eating away from home, such as at restaurants and cafes, and consuming convenient fast food, ready-to-eat (RTE) takeaway meals, and/or processed foods have been increasing in frequency due to lifestyle changes [1]. Consuming such foods is considered to be unhealthy because high frequencies of these meals and foods are associated with unbalanced nutrient intakes [2,3,4]. Furthermore, dietary ultra-processed food exposure is reportedly associated with adverse health outcomes, such as obesity, type 2 diabetes, cardiovascular disease, cancer, irritable bowel syndrome, depression, and frailty conditions, as well as increased cardiovascular and all-cause mortality [1,5]. The Japanese public has also shown an increased consumption of meals prepared outside the home, including processed and ready-to-eat foods [6,7], and these changes have made healthy eating difficult, leading to unbalanced nutrient intakes, especially for the younger generation [4,8,9,10,11]. There is sometimes little choice, when providing nutrition education to patients with lifestyle-related diseases who cannot cook on their own, but to recommend using RTE foods. However, few studies conducted in Japan have focused on the association between processed food consumption and biochemical parameters.

The NOVA food classification for processed food classifies all foods and food products into four groups according to the extent and purpose of the industrial processing they undergo. It considers all physical, biological, and chemical methods used during the food manufacturing processes, including the use of additives, and it has been used to study diet quality with highly processed foods being classified into group four as ultra-processed foods [12,13]. Various types of prepared RTE foods are sold in Japan. These are cooked and seasoned in various ways, including Westernized, Chinese, Ethnic and modern Japanese, and others; with fat and oil, such as fried, deep fried, and seasoned with mayonnaise, dressings, butter, and so on; or in more traditional Japanese ways without fat and oil, such as raw, boiled, simmered, stewed, grilled, and seasoned mainly with salt, sugar, vinegar, soy sauce, miso, and so on. Therefore, we divided foods not made at home according to their fat and oil content in order to evaluate diet quality.

It is noteworthy that increased ultra-processed food intake reportedly correlates with increased intakes of saturated fatty acids (SFA) and trans fat [2,3,14,15], and the excessive intakes of these fatty acids are risk factors for metabolic disorders, cardiovascular disease, and coronary heart disease [16,17,18,19]. Furthermore, the metabolism of fatty acids is affected not only by fatty acid intake itself but also impacts lipid accumulation in bodily tissues, leading to changes in circulating fatty acid composition [20,21]. However, the relationships among intakes of foods prepared outside of the home and circulating fatty acids have not been adequately investigated.

This study aimed to clarify the associations between intakes of processed and RTE foods with anthropometric and biochemical parameters, including circulating fatty acids, in Japanese residents of a metropolitan area.

## 2. Materials and Methods

### 2.1. Study Design and Participants

This was a cross-sectional study conducted according to the guidelines of the Declaration of Helsinki, and all procedures were approved by the Ethics Committee for Experimental Research Involving Human Subjects of Japan Women’s University (No. 265). We obtained written informed consent from all subjects prior to enrollment. The clinical trial registration number is UMIN000024195.

The participants were the same as those in our previous study, which was focused on non-alcoholic fatty liver disease in non-habitual drinkers [22]. However, in the present study, all participants were analyzed. The methods were described in detail in our previous report [22]. Japanese people who lived in the metropolitan area of Tokyo and 3 surrounding districts, 20 to 50 years of age, were recruited to participate in this study from 2016 to 2018. Patients who were taking medications, pregnant, nursing, engaged in high intensity exercise, and habitually consuming dietary supplements and/or health food products were excluded. Two hundred and thirteen subjects (109 men, 104 women) were enrolled, and all of their results were analyzed in this study.

### 2.2. Anthropometrics, Blood Pressure, and Biochemical Measurements

Anthropometric measurements and fasting blood collection were conducted at Japan Women’s University in the morning following a fast of at least 12 h. Body height and weight were measured, and body mass index (BMI) was calculated as weight (kg) divided by the square of height (m). Blood pressure was measured using an automatic blood pressure manometer with the participants in a seated position. Serum samples were obtained and stored at −80 °C until analyses. Measurements of total cholesterol, low-density lipoprotein cholesterol (LDL-C), high-density lipoprotein cholesterol (HDL-C), triglyceride (TG), phospholipids (PL), aspartate aminotransferase (AST), alanine aminotransferase (ALT), alkaline phosphatase (ALP), leucine aminopeptidase (LAP), γ-glutamyl transpeptidase (γ-GT), total bilirubin, and direct bilirubin were conducted at the Laboratory of BML Inc., Tokyo, Japan. The indirect bilirubin concentration was calculated from the total bilirubin minus the direct bilirubin concentration. The measurement methods used herein for each of these values were described in detail in our previous report [22].

### 2.3. Serum Elaidic Acid and Vaccenic Acid Analysis

Two 18:1 trans fatty acids, i.e., elaidic acid (18:1 trans-9) and vaccenic acid (18:1 trans-11), were measured at the Integrated Center for Mass Spectrometry, Kobe University Graduate School of Medicine. Fatty acid methylation and purification were performed using commercially available kits (Nacalai Tesque, Tokyo, Japan) according to the manufacturer’s protocol. Nonadecanoic acid (C19:0) was used as an internal standard. Fatty acid methyl esters were analyzed using gas chromatography–mass spectrometry (GC–MS QP2010-Ultra; Shimadzu, Kyoto, Japan). The capillary column used for fatty acid separation was SP-2650 (100 m × an inner diameter of 0.25 mm × membrane thickness of 0.20 μm, Sigma-Aldrich, St. Louis, MI, USA). The column temperature was maintained at 140 °C for 5 min, increased gradually by 1 °C/min to 240 °C, and held at this temperature for 20 min. The sample was injected in split mode with a split ratio of 1:5. The *m*/*z* 264 for ion monitoring was selected for the quantification of 18:1 trans fatty acids. All results were normalized to the peak height of the C19:0 internal standard.

### 2.4. Serum Fatty Acid Concentrations in PL Fraction

The fatty acid concentrations in serum PL were measured at the laboratory of Japan Women’s University. Serum lipids were extracted according to the method described by Folch et al. [23]. The lipid fractions were separated via thin-layer chromatography on silica gel with petroleum ether, diethyl ether, and acetic acid (84.15:15.0:0.85 *v*/*v*/*v*) as the solvent systems. The PL fraction was scraped into screw-cap glass tubes and methylated under argon gas in a heat bloc at 100 °C for 30 min with 3.5% sulfuric acid in methanol using C19:0 as an internal standard. The fatty acid methyl esters were separated via gas–liquid chromatography (Shimadzu GC14B, Tokyo, Japan) using a 0.25 × 30 m capillary column containing DB-FFAP (J&W Scientific, Santa Clara, CA, USA). The injector temperature was 230 °C, and the column temperature was increased in 2 °C/min increments from 160 °C to 200 °C. Nitrogen was used as the carrier gas, and the split ratio was 22.0. The coefficient of variation in each fatty acid lower than 5% was acceptable. Each fatty acid percent was calculated as its composition in PL. The C20:5(n3)(EPA)/C20:4(n6)(AA) ratio and each fatty acid percent were calculated.

### 2.5. Dietary Data Collection

The participants were asked to keep three-day (2 weekdays and 1 weekend day) dietary records during the week just before the examinations. They were asked to weigh and record all foods and beverages consumed on each day of recording. When dining out or eating take-out dishes on the go, the participants were instructed to take photos of the meals as well as the food and nutrient labels of the prepared foods they ate. The place of food consumption (home, restaurant, others), the means of obtaining the foods (home-made, takeout, dining out), as well as the product and company or store name for each of the processed foods were also recorded. The dietary records were confirmed and collected by research dietitians. Each ingredient and the weight of its consumed amount were estimated from portion sizes, ingredient labels, and nutritional information on the food packages, and query results and website information from the restaurants and manufacturers and cookbooks. Energy and nutrient intakes were calculated employing Excel-Eiyokun Version 8.0 software (Kenpakusha Co., Ltd., Tokyo, Japan) based on the Standard Tables of Food Composition in Japan 2015, seventh revised edition (Ministry of Education, Culture, Sports, Science and Technology, Tokyo, Japan). 

Processed foods, except drinks and dairy items, were categorized according to the requirements for both additional ingredients and cooking to make them edible, the need for additional cooking such as heating prior to consumption, and RTE foods and dishes. RTE foods and dishes were consumed outside of the home at a table (e.g., at a restaurant, café, fast-food shop), in the workplace, at a university, and at home. Furthermore, processed and RTE foods were divided into non-fatty or fatty foods according to fat and/or oil usage for processing and/or preparation (Table 1). 

For nutrients and food group intakes, the average intakes for three days were taken as the participants’ daily intakes. The food and nutrient intake amounts were adjusted by ideal body weight (IBW) in order to minimize the differences due to body size. The recommended BMI for Japanese individuals from 18 to 50 years of age ranges from 18.5 to 25, according to the Japan Atherosclerosis Society [24], though a BMI of 22 kg/m^2^ was regarded as corresponding to the hypothetical IBW [25,26] in this study. Physical activity was determined by employing a questionnaire to calculate energy expenditure [27].

### 2.6. Statistical Analysis

Statistical analyses were carried out using IBM SPSS Statistics (Version 27: IBM Japan Ltd., Tokyo, Japan). We used descriptive statistics with the means +/− standard deviation (SD) and median (interquartile range (IQR)), which are also presented for each continuous value. To detect group differences, the unpaired *t*-test and the Mann–Whitney test were applied to compare normally and non-normally distributed data, respectively. Each participant was grouped into one of the tertiles based on energy percent derived from fatty-RTE foods. The contributions of these fatty-RTE foods to nutrient intakes, biochemical parameters, and fatty acid compositions of serum PL were then evaluated. Trend associations across the tertiles were assessed by applying the Jonckheere–Terpstra test. A value of *p* < 0.05 was considered to indicate a statistically significant association.

## 3. Results

### 3.1. Characteristics of Participants

Most study participants were office worker (men 77.1%, women 53.8%), followed by smaller proportions of students and healthcare workers and teachers (men), part-time employees, homemakers and healthcare workers and teachers (women) (Table S1).

### 3.2. Energy Intakes from Processed and RTE Foods

The total energy intake for all participants was 31.6 ± 6.8 (mean ± SD) kcal/IBW. The respective median energy percents derived from processed and non-fatty RTE foods were 6.7 and 15.2, and the respective means of those from fatty-RTE foods and total processed and RTE foods were 36.4 and 63.2. Fatty-RTE foods were consumed more by men than by women (*p* < 0.001), while the energy intakes from processed and non-fatty-RTE foods did not differ between genders (Table S2).

### 3.3. Participant Backgrounds According to the Tertiles of Energy Percent Derived from Fatty-RTE Foods

The higher tertiles of energy derived from fatty-RTE foods included more men and younger participants (*p* < 0.001). The proportions of the participants differed among the tertiles with a larger percentage of office workers and students but fewer part-time employees and homemakers in the ascending order of the tertiles (*p* < 0.01) (Table 2).

### 3.4. Food Intakes According to the Tertiles of Energy Percent Derived from Fatty-RTE Foods

The energy percent from fatty-RTE food intakes showed strong negative associations with the intakes in grams per IBW of fish and fish products, soybeans and soybean products, dairy, eggs, green and yellow vegetables, other vegetables, seaweed + mushrooms + konjac, fruit, and non-oily seasonings (*p* < 0.01), while there were positive associations with fat/oil (*p* < 0.01), confectionaries, and sweet beverages (*p* < 0.05).

Consumptions of cereals, meat/poultry, processed meats, pickles, alcohol, and mayonnaise/dressing did not differ among the tertile groups (Table 3).

### 3.5. Nutrient Intakes According to the Tertiles of Energy Percent Derived from Fatty-RTE Foods

The median energy percent derived from fatty-RTE foods in the highest tertile was 54.7% with energy intake from fatty-RTE foods showing strong negative associations with those derived from processed foods requiring additional ingredients and/or cooking to make them edible (*p* < 0.001). Non-fatty RTE foods accounted for approximately 15% of energy, regardless of the fatty-RTE tertile. The energy derived from consuming fatty-RTE foods showed strong negative associations with dietary fiber, potassium, calcium, magnesium, β-carotene, vitamin K, vitamin B6, folate, pantothenic acid, biotin and ascorbic acid (*p* for trend < 0.001), protein, the sum of EPA and C22:6 (DHA), cholesterol, iron, selenium, retinol, vitamin D, alpha-tocopherol, thiamin, riboflavin, niacin, and vitamin B12 (*p* for trend < 0.01) and tended to be associated with carbohydrate (*p* = 0.056) (Table 4).

### 3.6. BMI, Blood Pressure, and Serum Biochemical Parameter Concentrations According to the Tertiles of Energy Percent Derived from Fatty-RTE Foods

Median (IQR) BMI, TG, and HDL-C were 21.4 (19.9, 23.3), 0.71 (0.52, 1.08) mmol/L, and 1.68 (1.42, 1.97) mmol/L, respectively, and mean LDL-C was 2.84 (0.75) (SD) mmol/L. BMI, blood pressure, serum LDL-C, serum TG, and liver function parameters were higher, but HDL-C and vaccenic acid levels were lower in men than in women (Table S3). Consuming fatty-RTE foods was positively associated with LAP (*p* for trend < 0.01), ALP, direct bilirubin, and elaidic acid (*p* for trend < 0.05) while being inversely associated with HDL-C (*p* for trend < 0.05) (Table 5).

### 3.7. Fatty Acid Compositions of PLs According to the Tertiles of Energy Percent Derived from Fatty-RTE Foods

The energy percent derived from consumption of fatty-RTE foods was negatively associated with C15:0, C17:0, DHA (*p* < 0.001), EPA (*p* < 0.01), AA, C18:3(n3), C20:4(n3), C22:5(n3) (*p* < 0.05), as well as the ratio of EPA/AA while being positively associated with C22:0, C24:0, and C18:2(n6) (*p* < 0.01) (Table 6).

## 4. Discussion

Our participants obtained approximately two-thirds of their total energy intake from processed and RTE foods, other than drinks and dairy items, thus exceeding the approximately one-third of total energy from highly/ultra-processed foods as reported in earlier Japanese studies using the NOVA system [9,10,11], suggesting that the consumption of highly processed food is rising in the younger generation in the metropolitan areas of Japan.

The dietary patterns in Japan have recently become highly diverse [28] due to increased consumption of various food types cooked with fat and oil. Traditionally, the Japanese people have eaten dishes cooked and seasoned without fat and oil. Thus, for this study, we divided RTE foods into those with and without fat and oil. While non-fatty RTE food accounted for approximately 15% of energy regardless of the fatty-RTE consumption level, processed foods used as ingredients in dishes were consumed less in the higher fatty-RTE food tertiles, suggesting that fatty-RTE consumption is lower for those eating homemade meals.

Concerning food group intake, fatty-RTE consumption reflected lower intakes of foods comprising main dishes, such as fish, eggs, and soy, and side dishes, such as vegetables, fruits, and seaweed/mushrooms/konjac. The Japan Atherosclerosis Society has recommended the Japan Diet (higher consumption of fish, soybeans and soy products, vegetables, seaweed/mushrooms/konjac, and unrefined cereals with reduced consumption of animal fat, fatty meat and poultry, sweets including desserts and snacks, and alcoholic drinks, along with reducing salt intake) as a preventive dietary pattern against atherosclerotic diseases [24,29,30]. Food consumptions in the higher tertile groups were the opposite of those recommended for adherence to the Japan Diet.

As for nutrient intake, we anticipated that a higher intake of fatty-RTE foods would correlate with higher fat and SFA intakes, as reported in previous studies conducted in other countries [1,2], but no differences were detected in either energy or fat intake, and the associations with protein and carbohydrate intakes were negative. A Japanese study using data from a survey on urban and suburban areas conducted in 2011 showed inverse relationships of the dietary share of ultra-processed foods with protein, vitamin K, vitamin B6, fiber, magnesium, phosphorous, and iron intakes [4]. Our results are consistent with those of this prior study, but our subjects showed insufficiencies in several additional minerals and vitamins. Furthermore, notably low intakes of functional nutrients, such as dietary fiber and EPA + DHA, were observed in the higher tertiles of fatty-RTE food intake in our present study.

Exposure to dietary ultra-processed foods is reportedly associated with obesity, hypertension, type 2 diabetes, and cardio-metabolic risk [5,31,32,33,34,35,36]. However, fatty-RTE intake was associated with only the lipid parameter of lower HDL-C in this study. These differences in health outcome parameters might be attributable to our participants having been relatively young and metabolically healthy with serum lipid levels being lower than those of subjects in the earlier studies, which were conducted in several countries. To our knowledge, there have been no reports documenting the association between the consumption of RTE foods and liver function parameters. The slight LAP and ALP elevations but no increases in AST, ALT, and γ-GT might suggest that our participants consuming rather high amounts of fatty-RTE were in the very early stages of liver fat accumulation. Cholestasis cannot, in our view, account for the small difference in bilirubin levels, which were within normal range.

This is the first study to focus on the association between circulating trans fats and food intakes in Japan. Circulating elaidic acid is a marker of industrial trans fatty acid (TFA) consumption from hydrogenated fat-containing products, such as margarine, shortenings [37], deep-fried foods [38], and ultra-processed foods [39]. Increased ultra-processed food intake reportedly correlates with increased trans fat intake [3,14,40], which leads to very damaging health consequences in terms of cardiovascular disease and cancer, more serious than those associated with SFA [16,17,18,19,38,39,40,41,42]. In 2018, the WHO called for the global elimination of industrially produced trans fat by 2023 and promulgated the REPLACE action framework with the aim of supporting countries in implementing best-practice policies [43]. In response, considerable efforts have been made to reduce the presence and dietary intakes of TFA in foods traditionally rich in TFA, such as bakery products, snacks, margarines, fats, and fast foods including those marketed by Japanese food companies. This study was conducted just before the beginning of the actions aimed at decreasing TFA in Japan. Japanese TFA intake was recognized as being lower than that in other countries [44], and serum elaidic acid concentrations were significantly lower in native Japanese than in Japanese–Americans [45]. However, the serum TFA level correlated positively with insulin resistance in the Japanese population [45] as well as with BMI, waist circumference, LDL-C, and TG while being inversely associated with HDL-C in young Japanese patients with coronary artery disease [46]. Compared with these studies, the elaidic aid concentration was very low herein, while a weak but statistically significant association was observed between serum elaidic aid and higher fatty-RTE food intake. A review suggested that more frequent consumption of fried foods is associated with negative health outcomes, such as developing type 2 diabetes, heart failure, obesity, and hypertension [38], and one of the reasons was suggested to be that oxidation during the process of frying food increases the amount of TFA in the food [47,48]. The fatty-RTE foods in this study consisted of deep-fried and stir-fried dishes, foods seasoned with mayonnaise and dressing, and fatty confectionaries. A limitation of this study is that we did not divide dishes according to the heating processes used. Further study is needed on the effects of RTE meal consumption, not only the types and volumes of fat and oil but also how the dishes are cooked, especially the means used to heat a meal, to ascertain the impacts on meal preparation outside of the home environment.

As to the fatty acid compositions in PLs, the most prominent trends, according to fatty-RTE food intakes, were the decreases in C15:0, C17:0, EPA, and DHA. C15:0 and C17:0 were previously reported to rise with the consumption of dairy products, but the concentrations of vaccenic acid derived from dairy products did not differ among the tertile groups and correlated positively with dairy intake (r = 0.294, *p* < 0.001) in this study. Recently, the circulating levels of odd-chain fatty acids have been noted to quantitatively reflect dietary fiber intake. High fiber consumption increases intestinal propionate formation by fermenting microbes [49,50], which are then converted to propionyl-CoA in organs that, in turn, competes with acetyl-CoA for condensation with malonyl-CoA such that C17:0 is formed instead of palmitate [51,52]. Propionate plays a role in reducing acetate utilization for fatty acid and cholesterol biosynthesis in the liver [52].

Maruyama and colleagues have documented C15:0 and C17:0 levels to be low in patients with metabolic syndrome [53]. Our group also reported that increased intakes of vegetables, seaweed/mushrooms/konjac, and unrefined grains can lead to increased dietary fiber consumptions in response to education on the Japan Diet, achieving reductions in body weight, serum LDL-C, and serum TG [54,55], which correlated with C15:0 and C17:0 increases in healthy participants and patients with dyslipidemia [21,56]. The results of this study are in line with those of previous investigations, suggesting that a higher consumption of fatty-RTE adversely impacts that of fiber rich foods. Education for processed food users is thus required with a focus on combining fiber-rich foods with fatty-RTE foods.

Since the end of the 20th century, fish intake in Japan has been decreasing annually, especially among young people [57]. Our study participants ranged from 20 to 50 years of age, and their fish intake was approximately identical to that of the same age group in the National Health and Nutrition Survey [58]. Circulating EPA, DHA, and C22:5 levels, markers of fish consumption [59,60], were lower than in our previous study on elderly subjects [21]. The highest tertile of fatty-RTE En% consumed only 15 g of fish (median), as reflected by the low EPA and DHA compositions of their PLs. On the other hand, the percent of C18:2(n6) in PLs was higher than previously reported in elderly subjects [21] and showed a positive association with fatty-RTE food intake, as reflected by a higher intake of dishes made using vegetable oils. Overconsumption of n-6 polyunsaturated fatty acids with a low intake of n-3 polyunsaturated fatty acids is highly associated with the pathogenesis of many diet-related chronic diseases such that investigators have focused on the n-6/n-3 polyunsaturated fatty acid ratio as it relates to diet for the past 40 years [61]. Polyunsaturated fatty acids metabolized via elongation and desaturation act as intermediates for various physiologically active substances, such as prostaglandins, thromboxanes, and leukotrienes [62,63], which are synthesized to serve as specialized pro-resolving lipid mediators, such as lipoxins from AA and resolvins, protectins, and maresins from EPA and DHA, during the initiation and the resolution phases of inflammation [64,65]. Consistent associations of a low circulating EPA/AA ratio with inflammation and chronic diseases have been reported [66,67], and these ratios have been demonstrated to serve as markers of atherosclerotic diseases [68,69]. It was recently reported that serum EPA/AA ratios decreased over a 10-year period, especially in middle-aged residents of a Japanese community, raising concern regarding a rise in future cardiovascular events [70,71]. Higher fatty-RTE food consumption was associated with a reduced EPA/AA ratio in PL, suggesting that growing consumption of RTE meals might be one of the reasons for negative changes in the circulating fatty acid profiles of Japanese people. Thus, it should be recommended that frequent consumers of fatty-RTE food consume more fish dishes.

This study has limitations. First, as our aim was to investigate the issues associated with the adverse impacts of consuming highly processed foods in Japan, we classified processed foods according to our original approach based on the fat and oil contents of such processed foods, without inclusion of drinks and dairy items, because most of these are used as ingredients in homemade dishes and desserts. Thus, we should meticulously compare the results with those obtained using the NOVA system. Second, we studied the participants according to the tertiles of energy percent derived from fatty-RTE foods, but there were obvious differences in age and gender. Further study is needed with a larger number of subjects, examining men and women separately. Third, though there are various types of TFAs, we measured serum elaidic and vaccenic acid levels, which are recognized as markers of industrial and ruminant TFA, respectively, such that we were unable to determine the significance of the TFA compositions of serum PLs. As trans fats are generated during food processing, measurements of the contents of the other TFAs are needed. Furthermore, differences among the means used to heat dishes should be analyzed in order to assess their effects on circulating TFA levels. Fourth, we were not able to determine TFA intakes because labeling of TFAs on commercial foods is not required in Japan. However, this is the first study to focus on processed food intake and metabolic parameters including serum fatty acids in Japan.

The demand for convenient foods may well rise further such that the consumption of highly processed foods is expected to increase in the future. We found that a high dependence on fatty-RTE food makes it difficult to consume the foods recommended for preventing arteriosclerosis and that the diets of individuals regularly eating these foods tend to be deficient in micronutrients, such as vitamins and minerals, and nutrients augmenting metabolic processes, such as fiber, EPA, and DHA. Fatty-RTE food intake was also suggested to be associated with an elevation of industrial TFAs in the blood, putting a strain on lipid metabolism, even in healthy participants not receiving medical care. If the younger generation continues to eat in this way well into the future, it may result in not only arteriosclerotic disease but also other health problems related to nutrient imbalances and deficiencies. It is necessary to improve the safety of processed foods and RTEs in various situations, such as by enhancing the quality of the ingredients and processing, as well as during product distribution, at sales venues, and by modifying the conditions of food preparation immediately prior to consumption. In addition, nutrition education on the advantages and disadvantages of processed foods is required to help consumers make better choices when consuming processed foods [72].

## 5. Conclusions

We studied the associations of processed food consumption with food and nutrient intake and health outcomes in Japanese individuals living in a metropolitan area. Men and younger participants consumed more fatty-RTE foods. A higher intake of fatty-RTE food was associated with lower intakes of foods prepared at home, which are comprised of traditional Japanese dietary ingredients, such as fish, soy, eggs, vegetables, fruits, and seaweed/mushrooms/konjac. The reduced intakes were reflected by insufficient nutrient intakes, including lower dietary fiber and EPA/DHA, leading to lower odd-numbered fatty acid and n-3 polyunsaturated fatty acid components in serum PL. Significant elevations of serum LAP, ALP, and elaidic acid with higher intakes of fatty-RTE food suggested the very early stages of excessive liver fat accumulation. Further large-scale studies are needed to update processed food consumption targets in the present highly diverse Japanese dietary environment. Efforts to improve the quality of processed and RTE foods as well as nutrition education, focused on maintaining a balanced diet, are also required.

## Figures and Tables

**Table 1:** Classification of processed foods.

	Requires Additional Cooking	Food Group	Made with Oil and/or Fat		Examples
Processed food	Requires additional ingredients and cooking	Canned and retort pouch foods	−		boiled tomatoes, fungi, beans
		Frozen foods	−		seafood mix, vegetables, edamame
		Seasonings, sauce	+		mayonnaise, dressing, pizza sauce
			−		jam, ketchup, soup for noodles
	Requires additional cooking, such as with heat	Cooked canned and bottled foods	+		boiled fish with vegetable oil
			−		boiled fish with dashi soup and soy sauce
		Cooked frozen foods	+		hamburger steak, pasta Bolognese, croquette
			−		stewed fish, Chinese dumplings, boiled fruits
		Cooked pouch foods	+		curry
			−		rice porridge
		Freeze-dried cooked foods	−		noodles, soup
		Other traditional prepared foods	+		fried tofu, fried onion
			−		konjac, mochi, Japanese noodles, pasta, kamaboko, hanpen, tofu, fu (Japanese dry baked wheat gluten), dried fruit, dried vegetables, nuts
Ready-to-eatfoods	Edible without cooking	Processed meat products	+	#	ham, sausage, bacon
		Sandwich, savory	+	#	sandwiches, hamburger, hot dog, pizza
		Eating at a restaurant or take-away staple and main dishes made with cereals, meat, chicken, eggs, fish, shellfish, and soybean products	+	#	ramen, fried rice, curry rice, tempura, pork cutlet, fried chicken
			−		rice ball, sushi, sashimi, natto, grilled fish
		Eating at a restaurant or take-away side dish and soup mainly made with vegetables, seaweed, fungi, soybean and soybean products, potatoes	+	#	stir-fried vegetables, salad with dressing or mayonnaise, fried potato, potage soup
			−		boiled or stewed vegetables seasoned with soy source or vinegar, tofu, baked potato, pickled vegetables, miso soup, clear soup
		Cereal flakes	−		corn flakes, muesli
		Plain table bread	−		bucket, table roll, bread
		Bakery products	+	#	Danish, sweet roll, doughnuts
		Confectionaries	+	#	pie, cookies, cakes, potato chips, ice cream, chocolate
			−		rice crackers, yokan, manjyu, sherbet, jelly

−: Without oil and/or fat, +: With oil and/or fat, #: Foods classified as fatty ready-to-eat (RTE) foods.

**Table 2:** Participant characteristics according to tertile of energy percent derived from fatty-RTE foods.

		T1			T2		T3		*p*	
			(n = 71)			(n = 71)		(n = 71)		
Age	(years)	41	(29, 47)	*	38	(31, 45)	32	(27, 41)	0.001	‡
Physical activity	(METs)	15.3	(12.3, 25.3)		14.5	(12.3, 25.3)	15.3	(12.3, 25.3)	0.197	‡
Men	(n)	23	(32.4)	†	41	(57.7)	45	(63.3)	<0.001	§
Occupation										
Office worker	(n)	41	(57.7)		49	(69.0)	53	(74.6)	0.006	
Part-time employee	(n)	10	(14.1)		3	(4.2)	1	(1.4)		
Homemaker	(n)	8	(11.3)		4	(5.6)	0	(0.0)		§
Healthcare worker and teacher	(n)	7	(9.9)		6	(8.5)	4	(5.6)		
Student	(n)	2	(2.8)		5	(7.0)	9	(12.7)		
Other	(n)	3	(4.2)		4	(5.6)	4	(5.6)		
Smoking states										
Never smoker	(n)	53	(74.6)		54	(76.1)	52	(73.2)	0.598	
Past smoker	(n)	9	(12.7)		13	(18.3)	11	(15.5)		§
Current smoker	(n)	9	(12.7)		4	(5.6)	8	(11.3)		

RTE: ready-to-eat. METs: metabolic equivalents. *: Values are presented as medians (IQR). †: %. ‡: *p* trend values were calculated using the Jonckheere–Terpstra test. §: *p* significance values for differences in proportions were calculated using the Chi-squared test.

**Table 3:** Food intake per ideal body weight according to tertile of energy percent derived from fatty-RTE foods.

	T1		T2		T3		*p*
	Median	(IQR)	Median	(IQR)	Median	(IQR)	
Cereal	6.480	(4.832, 7.944)	6.176	(4.810, 7.828)	6.224	(4.455, 7.217)	0.455
Potatoes and starch	0.467	(0.096, 0.836)	0.383	(0.092, 0.665)	0.312	(0.135, 0.706)	0.334
Sugar and jam	0.120	(0.061, 0.220)	0.098	(0.049, 0.165)	0.081	(0.037, 0.127)	0.006
Fish	0.589	(0.133, 0.853)	0.381	(0.076, 0.784)	0.116	(0.000, 0.547)	0.001
Seafood	0.044	(0.000, 0.276)	0.120	(0.000, 0.268)	0.033	(0.000, 0.217)	0.422
Soy and soybean products	0.770	(0.409, 1.469)	0.527	(0.228, 1.394)	0.280	(0.048, 0.820)	0.000
Dairy	1.731	(0.816, 3.125)	1.387	(0.556, 2.722)	0.373	(0.099, 1.268)	0.000
Meat and poultry	1.630	(1.092, 2.132)	1.879	(1.395, 2.382)	1.507	(1.040, 2.323)	0.645
Processed meat	1.664	(1.111, 2.256)	1.944	(1.395, 2.469)	1.508	(1.043, 2.402)	0.658
Eggs	0.596	(0.347, 0.872)	0.488	(0.225, 0.752)	0.339	(0.138, 0.658)	0.000
Fat and oil	0.186	(0.112, 0.257)	0.228	(0.156, 0.325)	0.267	(0.134, 0.356)	0.009
Fat	0.021	(0.000, 0.057)	0.023	(0.000, 0.078)	0.037	(0.014, 0.071)	0.072
Oil	0.149	(0.077, 0.233)	0.197	(0.119, 0.281)	0.206	(0.122, 0.309)	0.026
Mayonnaise, dressing	0.071	(0.025, 0.143)	0.114	(0.050, 0.179)	0.118	(0.033, 0.191)	0.118
Non-oily seasonings	0.688	(0.538, 0.946)	0.651	(0.518, 0.917)	0.587	(0.438, 0.725)	0.002
Nuts	0.004	(0.000, 0.033)	0.005	(0.000, 0.023)	0.009	(0.003, 0.037)	0.239
Green and yellow vegetables	1.348	(0.717, 2.698)	1.077	(0.607, 1.744)	0.808	(0.459, 1.139)	0.000
Other vegetables	2.458	(1.753, 3.531)	2.277	(1.605, 3.119)	1.607	(1.186, 2.298)	0.000
Pickles	0.037	(0.000, 0.184)	0.013	(0.000, 0.091)	0.053	(0.008, 0.115)	0.938
Seaweed, mushrooms, konjac	0.414	(0.191, 0.831)	0.287	(0.105, 0.471)	0.204	(0.082, 0.292)	0.000
Fruit	0.624	(0.000, 1.588)	0.216	(0.000, 1.059)	0.000	(0.000, 0.204)	0.000
Sweet beverages	0.000	(0.000, 1.569)	0.000	(0.000, 1.733)	0.893	(0.000, 2.880)	0.017
Confectionaries	0.478	(0.180, 0.994)	0.849	(0.336, 1.376)	0.805	(0.382, 1.357)	0.027
Alcohol	0.000	(0.000, 0.168)	0.000	(0.000, 0.241)	0.000	(0.000, 0.136)	0.604

The number of subjects is 71 in T1, T2, and T3. Values are expressed in grams per ideal body weight. RTE: ready-to-eat. *p* trend values were calculated using the Jonckheere–Terpstra test.

**Table 4:** Nutrient intakes per ideal body weight according to tertile of energy percent derived from fatty-RTE foods.

		T1		T2		T3		*p*
		Median	(IQR)	Median	(IQR)	Median	(IQR)	
Energy	(kcal)	32.1	(27.3, 38.0)	33.4	(29.0, 36.6)	29.1	(24.8, 34.5)	0.099
Processed food	(%En)	11.8	(5.4, 16.8)	7.4	(3.8, 12.0)	2.7	(0.4, 7.4)	0.000
Non-fatty RTE food	(%En)	14.6	(9.1, 23.0)	15.4	(9.5, 23.9)	16.3	(11.3, 23.2)	0.327
Fatty RTE	(%En)	17.9	(8.1, 23.5)	36.2	(33.1, 39.4)	54.7	(48.3, 64.5)	0.000
Protein	(g)	1.2	(1.0, 1.5)	1.2	(1.0, 1.4)	1.0	(0.8, 1.2)	0.002
Lipids	(g)	1.1	(0.9, 1.3)	1.2	(1.0, 1.4)	1.1	(0.9, 1.4)	0.836
SFA	(g)	0.325	(0.270, 0.402)	0.358	(0.305, 0.417)	0.307	(0.270, 0.412)	0.625
MUFA	(g)	0.383	(0.327, 0.492)	0.45	(0.362, 0.548)	0.414	(0.324, 0.522)	0.535
PUFA	(g)	0.217	(0.178, 0.268)	0.23	(0.192, 0.273)	0.221	(0.162, 0.282)	0.815
n-6 PUFA	(g)	0.19	(0.15, 0.22)	0.19	(0.16, 0.22)	0.18	(0.14, 0.24)	0.945
n-3 PUFA	(g)	0.04	(0.03, 0.05)	0.03	(0.03, 0.05)	0.03	(0.02, 0.04)	0.295
EPA + DHA	(mg)	9.74	(2.46, 17.42)	7.14	(2.95, 14.69)	2.87	(1.27, 9.23)	0.009
Cholesterol	(mg)	5.73	(4.24, 7.04)	5.28	(4.13, 6.65)	4.54	(3.12, 5.97)	0.002
Carbohydrate	(g)	3.95	(3.34, 4.84)	3.78	(3.42, 4.50)	3.54	(2.98, 4.31)	0.056
Dietary fiber	(g)	0.24	(0.19, 0.31)	0.21	(0.16, 0.26)	0.17	(0.15, 0.22)	0.000
Sodium	(mg)	57.1	(48.6, 68.7)	57.1	(49.4, 64.9)	56.5	(44.8, 63.8)	0.414
Potassium	(mg)	41.5	(34.1, 54.0)	39.0	(31.8, 45.0)	31.6	(25.6, 38.5)	0.000
Calcium	(mg)	8.5	(6.8, 10.8)	7.1	(5.6, 9.6)	6.0	(4.3, 7.6)	0.000
Magnesium	(mg)	4.3	(3.6, 5.4)	4.2	(3.5, 4.8)	3.3	(2.8, 4.3)	0.000
Iron	(mg)	0.14	(0.11, 0.16)	0.12	(0.10, 0.14)	0.10	(0.08, 0.13)	0.001
Iodine	(μg)	13.8	(2.8, 28.5)	14.3	(4.2, 29.9)	14.6	(2.8, 24.9)	0.678
Selenium	(μg)	1.2	(0.9, 1.5)	1.2	(0.9, 1.4)	1.0	(0.8, 1.4)	0.011
β-carotene	(μg)	59.1	(35.6, 89.1)	48.2	(28.1, 65.4)	34.5	(22.2, 56.3)	0.000
Retinol	(μg)	8.2	(6.0, 11.4)	7.2	(4.8, 10.2)	5.7	(3.9, 8.5)	0.002
Vitamin D	(μg)	0.10	(0.04, 0.17)	0.08	(0.04, 0.14)	0.05	(0.03, 0.09)	0.001
α-tocopherol	(mg)	0.13	(0.11, 0.16)	0.12	(0.10, 0.15)	0.11	(0.09, 0.13)	0.002
Vitamin K	(mg)	4.10	(2.49, 6.63)	3.45	(2.19, 4.37)	2.39	(1.89, 3.48)	0.000
Thiamin	(mg)	0.02	(0.01, 0.02)	0.02	(0.01, 0.02)	0.01	(0.01, 0.02)	0.005
Riboflavin	(mg)	0.02	(0.02, 0.03)	0.02	(0.02, 0.02)	0.02	(0.01, 0.02)	0.001
Niacin	(mg)	0.53	(0.44, 0.67)	0.54	(0.43, 0.62)	0.43	(0.34, 0.52)	0.001
Vitamin B6	(mg)	0.02	(0.02, 0.03)	0.02	(0.02, 0.03)	0.02	(0.01, 0.02)	0.000
Vitamin B12	(mg)	0.09	(0.05, 0.17)	0.11	(0.06, 0.14)	0.06	(0.03, 0.09)	0.019
Folate	(mg)	5.33	(4.11, 7.52)	4.71	(3.67, 6.12)	3.84	(2.83, 5.11)	0.000
Pantothenic acid	(mg)	0.10	(0.08, 0.13)	0.10	(0.08, 0.12)	0.08	(0.06, 0.10)	0.000
Biotin	(mg)	0.68	(0.50, 0.80)	0.58	(0.49, 0.71)	0.49	(0.36, 0.59)	0.000
Ascorbic acid	(mg)	1.61	(1.00, 2.32)	1.42	(0.98, 1.91)	1.09	(0.81, 1.54)	0.001

The number of subjects is 71 in T1, T2, and T3. Values are presented as medians (IQR). Values are expressed per ideal body weight (kg). *p* trend values were calculated using the Jonckheere–Terpstra test. Processed food: processed food requires additional ingredients and cooking. RTE: ready-to-eat, SFA: saturated fatty acid, MUFA: monounsaturated fatty acid, PUFA: polyunsaturated fatty acid, EPA: icosapentaenoic acid, DHA: docosahexaenoic acid.

**Table 5:** Body mass index, blood pressure, serum biochemical parameters, and trans fatty acid concentrations according to tertile of energy percent derived from fatty-RTE foods.

		T1		T2		T3		*p*
		Median	(IQR)	Median	(IQR)	Median	(IQR)	
Body weight	(kg)	54.2	(49.0, 63.1)	58.4	(52.0, 68.0)	61.7	(54.3, 70.1)	0.002
Body mass index	(kg/m^2^)	21.0	(19.7, 22.8)	21.4	(19.8, 23.6)	21.9	(20.3, 23.6)	0.100
Systolic blood pressure	(mmHg)	109	(102, 119)	114	(107, 129)	114	(102, 128)	0.073
Diastolic blood pressure	(mmHg)	70	(64, 77)	72	(65, 80)	72	(63, 80)	0.536
Total cholesterol	(mmol/L)	4.91	(4.45, 5.48)	5.22	(4.47, 5.61)	5.02	(4.47, 5.53)	0.816
LDL cholesterol	(mmol/L)	2.71	(2.17, 3.34)	2.87	(2.30, 3.36)	2.87	(2.46, 3.47)	0.129
HDL cholesterol	(mmol/L)	1.73	(1.50, 2.07)	1.68	(1.32, 1.99)	1.55	(1.40, 1.86)	0.033
Triglyceride	(mmol/L)	0.71	(0.59, 0.96)	0.66	(0.49, 1.08)	0.75	(0.52, 1.26)	0.378
Phospholipid	(mmol/L)	27.4	(25.3, 30.2)	28.0	(25.7, 31.1)	27.3	(24.2, 29.6)	0.732
Aspartate aminotransferase	(U/L)	19	(17, 24)	20	(18, 22)	20	(18, 25)	0.609
Alanine aminotransferase	(U/L)	16	(12, 23)	16	(12, 21)	19	(12, 26)	0.135
Alkaline phosphatase	(U/L)	159	(135, 199)	192	(154, 213)	179	(154, 219)	0.010
γ-glutamyl transpeptidase	(U/L)	19	(14, 33)	20	(15, 28)	23	(14, 39)	0.261
Leucine aminopeptidase	(U/L)	46	(42, 54)	49	(43, 56)	52	(46, 59)	0.001
Total bilirubin	(µmol/L)	12.0	(8.5, 15.4)	12.0	(10.3, 13.7)	13.7	(10.3, 15.4)	0.142
Direct bilirubin	(µmol/L)	3.4	(3.4, 5.1)	3.4	(3.4, 5.1)	5.1	(3.4, 5.1)	0.019
Indirect bilirubin	(µmol/L)	8.5	(6.8, 10.3)	8.5	(6.8, 10.3)	8.5	(6.8, 10.3)	0.380
Elaidic acid	(μM)	3.51	(3.06, 4.24)	3.78	(3.23, 4.71)	3.86	(3.30, 4.73)	0.021
Vaccenic acid	(μM)	4.64	(3.55, 6.24)	4.45	(3.43, 5.46)	4.26	(3.37, 5.57)	0.362

The number of subjects is 71 in T1, T2, and T3. *p* trend values were calculated using the Jonckheere–Terpstra test. RTE: ready-to-eat.

**Table 6:** Phospholipid fatty acid compositions according to tertile of energy percent derived from fatty-RTE foods.

	T1		T2		T3		*p*
	Median	(IQR)	Median	(IQR)	Median	(IQR)	
SFA 12:0	0.01	(0.00, 0.01)	0.01	(0.00, 0.01)	0.01	(0.01, 0.01)	0.860
14:0	0.26	(0.18, 0.31)	0.26	(0.16, 0.32)	0.22	(0.15, 0.31)	0.085
15:0	0.12	(0.10, 0.14)	0.11	(0.09, 0.13)	0.10	(0.09, 0.12)	0.000
16:0	24.98	(24.16, 26.13)	25.08	(24.06, 26.1)	25.04	(24.47, 25.88)	0.625
17:0	0.36	(0.32, 0.4)	0.34	(0.30, 0.37)	0.33	(0.30, 0.35)	0.000
18:0	13.89	(13.21, 14.36)	13.87	(13.11, 14.65)	13.74	(13.2, 14.62)	0.929
20:0	0.61	(0.55, 0.68)	0.60	(0.54, 0.66)	0.62	(0.56, 0.70)	0.301
22:0	1.48	(1.30, 1.67)	1.50	(1.32, 1.75)	1.67	(1.44, 1.81)	0.002
24:0	1.32	(1.16, 1.49)	1.36	(1.19, 1.53)	1.47	(1.30, 1.59)	0.007
MUFA 16:1	0.35	(0.30, 0.41)	0.34	(0.29, 0.44)	0.34	(0.28, 0.41)	0.577
17:1	0.09	(0.08, 0.12)	0.09	(0.07, 0.12)	0.09	(0.07, 0.12)	0.351
18:1cis	8.11	(7.71, 8.68)	8.28	(7.69, 9.08)	8.45	(7.77, 9.23)	0.095
20:1	0.22	(0.19, 0.26)	0.23	(0.20, 0.27)	0.22	(0.19, 0.24)	0.800
22:1	0.04	(0.04, 0.05)	0.04	(0.03, 0.05)	0.04	(0.03, 0.05)	0.990
24:1	2.71	(2.51, 2.95)	2.75	(2.38, 3.02)	2.84	(2.50, 3.11)	0.418
n-6 PUFA 18:2cis	20.03	(17.98, 21.84)	20.61	(18.98, 21.9)	21.58	(19.58, 23.41)	0.001
20:2	0.33	(0.29, 0.37)	0.31	(0.28, 0.35)	0.32	(0.28, 0.36)	0.709
20:3	1.98	(1.54, 2.37)	1.99	(1.64, 2.35)	1.92	(1.69, 2.42)	0.847
(AA) 20:4	10.30	(9.43, 11.6)	10.17	(8.88, 10.79)	9.78	(8.82, 11.11)	0.027
22:2	0.64	(0.53, 0.69)	0.58	(0.52, 0.68)	0.62	(0.53, 0.70)	0.508
n-3 PUFA 18:3	0.22	(0.20, 0.27)	0.21	(0.17, 0.25)	0.21	(0.16, 0.27)	0.031
18:4	0.09	(0.07, 0.13)	0.09	(0.07, 0.12)	0.09	(0.07, 0.12)	0.774
20:4	0.10	(0.07, 0.16)	0.10	(0.07, 0.14)	0.09	(0.06, 0.12)	0.013
(EPA) 20:5	1.86	(1.10, 2.53)	1.71	(1.06, 2.47)	1.14	(0.72, 1.77)	0.001
22:5	0.97	(0.86, 1.11)	0.93	(0.80, 1.05)	0.88	(0.79, 1.01)	0.013
(DHA) 22:6	6.71	(5.72, 7.48)	6.07	(5.11, 6.98)	5.83	(4.75, 6.71)	0.000
EPA/AA	0.182	(0.106, 0.244)	0.182	(0.102, 0.256)	0.115	(0.078, 0.183)	0.003

The number of subjects is 71 in T1, T2, and T3. Values are expressed as percent of total fatty acids in phospholipids. RTE: ready-to-eat, SFA: saturated fatty acid, MUFA: monounsaturated fatty acid, PUFA: polyunsaturated fatty acid. *p* trend values were calculated using the Jonckheere–Terpstra test.

## Data Availability

Data are contained within the article and Supplementary Materials.

## Supplementary Materials

The following supporting information can be downloaded at: https://www.mdpi.com/article/10.3390/nu16071032/s1, Table S1: Characteristics of participants; Table S2: Energy intake from processed and RTE foods; Table S3: Parameters of participants.
