# Supplementary material for: A Cross-Sectional Pilot Study on Association of Ready-to-Eat and Processed Food Intakes with Metabolic Factors, Serum Trans Fat and Phospholipid Fatty Acid Compositions in Healthy Japanese Adults
Source: Nutrients. 2024 Apr 2;16(7):1032. doi: 10.3390/nu16071032 (PMC11013905; doi:10.3390/nu16071032)
Supplement: Supplementary file 1 [file nutrients-16-01032-s001.zip › nutrients-2901863-supplementary.pdf]

Table S1. Characteristics of participants

		All (n＝213)			Men (n=109)			Women (n=104)			*p*
Age	(y)	37	±	9 *	35	±	9	38	±	9	0.028
Occupation											<0.001
Full-time employee	(%)	65.7			77.1			53.8			
Part-time employee	(%)	8.0			0.0			16.3			
Homemaker	(%)	5.6			0.0			11.5			
Healthcare worker and teacher	(%)	8.0			4.6			11.5			
Student	(%)	7.5			11.0			3.8			
Other	(%)	5.2			7.3			2.9			
Smoking habits											0.008
Non-smoker	(%)	74.6			67.0			82.7			
Past-smoker	(%)	15.5			17.4			13.5			
Smoker	(%)	9.9			15.6			3.8			
Physical activity	(METs)	18.2	±	7.3 *	17.7	±	7.4	18.6	±	7.3	0.369

*: Values are presented as mean ±standard deviation.

p values are calculated using the unpaired t-test for comparison between men and women, and using the Chi-squared test of independence for distribution comparison between men and women.

METs: Metabolic equivalents

Table S2. Energy intake from processed and RET food

		All (n=213)		Men (n=109)		Women (n=104)		p
Total energy intake	(kcal/ IBW)*	31.6	(6.8)	32.0	(6.7)	31.2	(6.9)	0.000
Processed food [a]	(kcal /IBW)	2.1	(0.9 , 3.9)	1.88	(0.64 , 3.87)	2.36	(1.03 , 4.10)	0.150
Non-fatty RTE food	(kcal /IBW)	5.0	(3.2 , 7.3)	5.43	(3.58 , 7.50)	4.72	(2.64 , 6.99)	0.170
Fatty-RTE food	(kcal /IBW)*	11.3	(6.0)	12.94	(6.1)	9.51	(5.39)	0.000
RTE food	(kcal /IBW)*	16.8	(7.3)	18.87	(7.70)	14.73	(6.22)	0.000
Processed and RTE food	(kcal /IBW)*	19.7	(6.7)	21.56	(6.99)	17.82	(5.80)	0.000
Processed food [a]	(%En)	6.7	(2.7 , 12.5)	5.72	(1.69 , 12.62)	7.28	(3.08 , 12.37)	0.136
Non-fatty-RTE food	(%En)	15.2	(9.6 , 23.2)	15.34	(10.51 , 23.03)	15.05	(8.89 , 23.29)	0.352
Fatty-RTE food	(%En)*	36.4	(18.7)	40.80	(18.43)	31.78	(18.01)	0.000
RTE food	(%En)	53.2	(37.9 , 71.5)	59.19	(42.14 , 78.09)	47.55	(35.17 , 62.94)	0.000
Processed and RTE food	(%En)*	63.2	(18.8)	68.13	(19.66)	58.09	(16.44)	0.000

Values are presented as medians (IQR). *: Values are presented as means (SD).

a: processed food require additional ingredients and/or cooking.

RTE: ready to eat, IBW: ideal body weight.

Table S3. Parameters of participants

		All (n＝213)		Men (n=109)		Women (n=104)		p
Age		37.0	(28.0 , 44.5)	34.0	(27.5 , 43)	39.5	(30.0 , 47.0)	0.031
Body weight	(kg)	58.2	(51.6 , 67.3)	66.4	(61.0 , 73.3)	51.8	(48.1 , 55.9)	<0.001
Body mass index	(kg/m²)	21.4	(19.9 , 23.3)	22.5	(20.9 , 24.2)	20.6	(19.4 , 21.8)	<0.001
Systolic blood pressure	(mmHg)	112	(104 , 125)	122	(111 , 132)	106	(99 , 112)	<0.001
Diastolic blood pressure	(mmHg)	71	(64 , 78)	75	(68 , 85)	67	(62 , 74)	<0.001
Total-cholesterol*	(mmol/L)	5.07	(0.88)	5.07	(0.93)	5.04	(0.80)	0.841
LDL-cholesterol*	(mmol/L)	2.84	(0.75)	2.95	(0.72)	2.74	(0.72)	0.034
HDL-cholesterol	(mmol/L)	1.68	(1.42 , 1.97)	1.45	(1.27 , 1.68)	1.86	(1.68 , 2.07)	<0.001
Triglyceride	(mmol/L)	0.71	(0.52 , 1.08)	0.91	(0.64 , 1.71)	0.60	(0.46 , 0.79)	<0.001
Phospholipid	(mmol/L)	27.5	(25.3 , 30.2)	27.4	(24.5 , 30.2)	27.8	(25.7 , 30.5)	0.275
Aspartate aminotransferase	(U/L)	20	(18 , 24)	22	(18 , 26)	19	(16 , 21)	<0.001
Alanine aminotransferase	(U/L)	16	(12 , 24)	20	(16 , 29)	14	(11 , 17)	<0.001
Alkaline phosphatase	(U/L)	175	(148 , 212)	200	(165 , 234)	157	(131 , 179)	<0.001
γ-glutamyl transpeptidase	(U/L)	20	(15 , 33)	26	(18 , 39)	16	(12 , 24)	<0.001
Leucine aminopeptidase	(U/L)	49	(44 , 56)	54	(49 , 61)	45	(41 , 49)	<0.001
Total bilirubin	(μmol/L)	12.0	(10.3 , 15.4)	13.7	(10.3 , 15.4)	12.0	(8.5 , 13.7)	0.003
Direct bilirubin	(μmol/L)	3.4	(3.4 , 5.1)	5.1	(3.4 , 5.1)	3.4	(3.4 , 3.4)	<0.001
Indirect bilirubin	(μmol/L)	8.5	(6.8 , 10.3)	8.5	(6.8 , 10.3)	8.5	(6.8 , 10.3)	0.063
Elaidic acid	(μM)	3.740	(3.245 , 4.605)	3.760	(3.245 , 4.72)	3.715	(3.238 , 4.308)	0.550
Vaccenic acid	(μM)	4.470	(3.42 , 5.715)	4.060	(2.940 , 5.205)	5.035	(3.865 , 6.223)	<0.001

Values are presented as medians (IQR). *: Values are presented as means (SD).
